# Drug Development Strategies for Malaria: With the Hope for New Antimalarial Drug Discovery—An Update

**DOI:** 10.1155/2023/5060665

**Published:** 2023-03-14

**Authors:** Swaroop Kumar Pandey, Uttpal Anand, Waseem A. Siddiqui, Renu Tripathi

**Affiliations:** ^1^Department of Life Sciences, The National Institute for Biotechnology in the Negev, Ben-Gurion University of the Negev, Beer-Sheva 84105, Israel; ^2^Department of Life Sciences, Ben-Gurion University of the Negev, Beer-Sheva 84105, Israel; ^3^Interdisciplinary Biotechnology Unit, Aligarh Muslim University, Aligarh 202001, Uttar Pradesh, India; ^4^Department of Molecular Microbiology and Immunology, CSIR-Central Drug Research Institute, Lucknow 226031, Uttar Pradesh, India

## Abstract

Malaria continued to be a deadly situation for the people of tropical and subtropical countries. Although there has been a marked reduction in new cases as well as mortality and morbidity rates in the last two decades, the reporting of malaria caused 247 million cases and 619000 deaths worldwide in 2021, according to the WHO (2022). The development of drug resistance and declining efficacy against most of the antimalarial drugs/combination in current clinical practice is a big challenge for the scientific community, and in the absence of an effective vaccine, the problem becomes worse. Experts from various research organizations worldwide are continuously working hard to stop this disaster by employing several strategies for the development of new antimalarial drugs/combinations. The current review focuses on the history of antimalarial drug discovery and the advantages, loopholes, and opportunities associated with the common strategies being followed for antimalarial drug development.

## 1. Introduction

Malaria continues to be a major public health concern and has a significant global impact. Malaria is a vector-borne disease caused by certain species of unicellular eukaryote *Plasmodium* spp. [1]. However, *P. falciparum* infection is mainly responsible for most of the malaria-related morbidity and mortality, mostly among African children and pregnant women. WHO and other independent research organizations worldwide are continuously working to manage this lethal infection [2, 3]. There are two main strategies for the management and control of malaria infection. The first is to control the vector, and the second is to manage the infected cases [4, 5]. However, several agencies are also involved in the development of an effective vaccine. The most common vaccine, RTS, S has promising results. However, it has no effect on transmission, hence endemicity [6].

Management of infected cases basically relies on antimalarial drugs/combinations. However, the development of drug resistance and cross-resistance against most of the antimalarials (including atovaquone, sulfadoxine, pyrimethamine, and mefloquine, and even more recently the most efficacious artemisinin derivatives) and the declining efficacy of combinations in clinical practice is a big hurdle to case management [7, 8]. Therefore, there is an urgent need for the development of new therapeutic agents/drug combinations, which are effective in tackling drug resistance and have higher efficacy with faster action for the treatment of malaria, especially in developing countries. Antimalarial drug discovery can follow several strategies, ranging from minor modifications of existing agents to the design of novel agents that act against new targets. This article will discuss some common and important strategies for antimalarial drug discovery and the major associated challenges.

## 2. History of Drug Development for Malaria

Cinchona bark was the first effective antimalarial used in the seventeenth century, and its active ingredient, quinine, was isolated in 1820. Quinine became a treatment of reference for intermittent fever throughout the world, and it is still an important and effective treatment for malaria. The actual action mechanism of quinine remains controversial, but it is hypothesized that it acts on the asexual stage of the malaria parasite by inhibiting its heme polymerase enzyme, thereby inhibiting hemozoin formation, an essential process for the survival of the malaria parasite [9].

The first synthetic antimalarial drug, pamaquine, was developed in 1925 by German researchers by modifying methylene blue. Due to its low efficacy and high toxicity, pamaquine cannot be used for the treatment of malaria. But it provided lead compounds for the development of better antimalarial agents like mepacrine (quinacrine) [10].

Chloroquine (CQ) was first synthesized in Germany by Bayer Corporation in 1934 as a cheaper alternative to the costly naturally occurring quinine, but it was then considered toxic for any significant biological use [11]. However, in the late 1950s, a high level of resistance against CQ was reported from several parts of tropical and subtropical regions. The proposed action mechanism of CQ is the same as that of quinine [12].

Proguanil, a pyrimidine derivative and folate pathway inhibitor, was also introduced during World War II. Proguanil is currently being used as a fixed-dose combination with atovaquone for treatment and chemoprophylaxis agents for preventing malaria in travelers. It is an inhibitor of dihydrofolate reductase (DHFR) [13].

Atovaquone is a hydroxynaphthoquinone drug active against all *Plasmodium* species. Atovaquone acts by inhibiting parasite mitochondrial electron transport as it is an analogue of ubiquinone, a parasite mitochondrial electron carrier, which is the cofactor of dihydroorotate dehydrogenase [14]. The atovaquone-proguanil combination is well more effective than CQ alone, CQ-SP, or mefloquine (MQ) against multidrug-resistant *P. falciparum*. It is also effective in proguanil-resistant regions [15].

Pyrimethamine, which belongs to the same chemical class as proguanil, was further developed and is now being used as a fixed-dose combination with sulphadoxine (a sulfonamide and the structural analogues and competitive antagonists of p-aminobenzoic acid) for uncomplicated malaria since the late 60s. The sulphadoxine-pyrimethamine (SP) combination is commonly called Fansidar. Resistance to SP in Africa remained low until the late 1990s, but since then it has spread rapidly. The combination works by inhibiting two important enzymes of the folate pathway, dihydropteroate synthase (DHPS) and DHFR, respectively [16].

In the late 80s, MQ was developed as an effective treatment option for uncomplicated malaria through a collaborative project of the US Army Medical Research and Development Command, WHO/TDR, and Hoffman-La Roche, Inc [17]. MQ is an effective blood schizontocidal against all malaria species that infect humans, including the fifth species, *Plasmodium knowlesi*. Resistance to mefloquine was reported in Asia in 1985, around the time the drug became generally available. The exact mode of antimalarial action and biochemical basis of resistance to MQ are not known, but clinical resistance to MQ is associated primarily with the amplification of the pfMDR1 gene, which encodes a P-glycoprotein homologue1. MQ inhibits *β*-haematin formation, leading to a toxic accumulation of haem (ferriprotoporphyrin IX) in the parasite's food vacuole [18].

Another important antimalarial drug, piperaquine (PPQ), was synthesized by the Shanghai Research Institute of Pharmaceutical Industry in 1966 and was used as a replacement for CQ as the first-line monotherapy in China for malaria treatment [19]. The exact mechanism of action of PPQ is unknown. But it has been shown that it acts on the polymerization of haematin. The exact mechanism of resistance against PPQ is not well known yet; however, a copy number variation event on chromosome 5 of the malaria parasite was observed in PPQ resistance [20, 21].

Lumefantrine (LMF) was synthesized originally by the Academy of Military Medical Sciences in Beijing, China. The structure and mode of action are similar to those of QN and MQ. LMF is now recommended in combination with fast-acting artemisinin derivatives, such as artemether. In this combination, LMF is responsible for eliminating the residual parasites [22].

Pyronaridine, a benzonaphthyridine, was synthesized in China in 1970 [23]. The action mechanism of pyronaridine is similar to that of CQ [23]. Pyronaridine has been used extensively as monotherapy to treat *P. falciparum* and *P. vivax* infections by oral and parenteral routes in Hunan province. The action and resistance mechanism of pyronaridine are similar to those of CQ, but it has better antimalarial potential than CQ [24].

Artemisinin (ART) and its derivatives are derived/obtained from the plant *Artemisia annua*. *Artemisia annua*, an annual herb belonging to the *Asteraceae* family, has been used as an antimalarial herb in China for over 1000 years. *Artemisia annua*, also known as sweet wormwood, sweet annie, sweet sagewort, annual wormwood, or qinghao, is a common type of wormwood that is native to temperate Asia (mainly northern parts of China) but naturalized throughout the world. In 1971, the antimalarial activity of plant extract was experimentally proved in a primate model, and in 1972, ART was isolated and its chemical structure was described [24].

ART is an endoperoxide sesquiterpene lactone produced by the aerial parts (leaves) of *Artemisia annua* L. and is effective even against multidrug-resistant strains of the malaria parasite. Several derivatives of ART have been developed by semisynthetic substitutions to enhance the pharmacological profile of ART. ART and its derivatives are potent blood schizontocidal and gametocytocidal compounds that can cure infected patients and also act as transmission-blocking agents [24]. The presence of the endoperoxide bridge is the key factor and is responsible for the potent antimalarial potential of this class [25]. It is supposed that ART derivatives perform their action through the production of free radicals and reactive aldehydes [26]. ARTs perform their antimalarial action in two successive steps. The first step is the iron-mediated cleavage of the endoperoxide bridge, which generates an unstable organic free radical and/or other electrophilic species. The second step of alkylation involves the formation of covalent adducts between the drug and malarial proteins [26]. All the ART derivatives have specific physicochemical as well as biological properties. The main ART derivatives are artemether, arteether (AE), dihydroartemisinin (DHA), and artesunate (AS). Artemether (AM) is a methyl ether derivative of dihydroartemisinin, and it is synthesized by the reduction of dihydroartemisinin. AM can be given as an oil-based intramuscular injection or orally. It is also coformulated with LMF for combination therapy [27]. *α*/*β* arteether is an ethyl ether derivative of artemisinin. It is a potent, oil-soluble antimalarial. AE was developed by the CSIR-Central Drug Research Institute (CDRI), Lucknow, India, and consists of a mixture of *α* and *β* forms of AE in a 30 : 70 ratio. It was registered in India in January 1997 for use in malaria patients. This drug was marketed in India as an oil-based intramuscular injectable formulation [28]. Dihydroartemisinin (DHA) is the main active metabolite of the ART derivatives. It is relatively insoluble in water. Its antimalarial potential is similar to that of oral AS [29]. It can be given orally as well as by the rectal route. A fixed-dose formulation with PPQ [30]is currently being used as a promising new ART-based combination treatment (ACT). It is the most effective ART compound, has a strong blood schizonticidal action, and reduces malaria transmission. Artesunate (AS) is the sodium salt of the hemisuccinate ester of ART. It is soluble in water as well as in alkaline water but has poor stability in aqueous solutions at neutral or acidic pH. AS can be given orally, rectally, or by the intramuscular or intravenous routes [31]. A combination of MQ and AS is highly effective in treating multidrug-resistant malaria. Researchers are continuously working to find some new, potent, and safe antimalarial agents. Several new antimalarials and/or combinations are in different developmental stages and may be ready for use in the future for malaria patients (Table 1).

## 3. The Challenges with Antimalarials

Resistance to antimalarial drugs hampers control efforts and increases the risk of morbidity and mortality from malaria. Antimalarial drug resistance has been defined as the ability of a parasite strain to survive and/or multiply despite the administration and absorption of a drug given in doses equal to or higher than those usually recommended but within the tolerance of the subject [32]. In general, drug resistance appears to occur through spontaneous mutations in the target genes of the malaria parasite and is thought to be independent of the drug used. It may be a single mutation or multiple mutations. These genetic mutations in the genes responsible for producing the proteins related to the drug's parasite target or influx/efflux pumps lead to a reduction of intraparasitic concentrations of the drug. For example, mutations in the genes encoding the transporters PfCRT and PfMDR1 are responsible for CQ drug resistance and cross resistance to closely related drugs. However, the role of mutations in PfMDR1 in determining the therapeutic response following chloroquine treatment remains unclear. There are several other examples, like single-point mutations in the gene encoding cytochrome b (cytB), which confer atovaquone resistance, or in the gene encoding dihydrofolate reductase (dhfr), responsible for pyrimethamine resistance [33]. Immunity also plays a major role in the emergence and spread of drug resistance. Even a previously nonimmune individual develops a specific immune response to a malaria infection, and eventually the malaria parasite evades this response by programming antigenic variation in its main red cell surface-expressed epitopes. For example, *P. falciparum* infects erythrocyte membrane protein 1 (PfEMP1), which is encoded by the var multigene family and changes in 2-3% of parasites each asexual cycle. Drug resistance also depends on transmission in geographical areas, immunity, parasite load, and the PK-PD properties of the drug. Drugs with long half-lives, for which resistance is conferred by single-point mutations, select resistant parasites rapidly. Uncontrolled use and poor-quality or fake drugs also contribute to the emergence of resistance [32–35].

## 4. Important Strategies for Antimalarial Drug Discovery

### 4.1. Developing Analogues of Existing Drugs

During the last two decades, only a few chemical series have been identified and are in clinical practice as a new class of antimalarial drugs, including aminoalcohols (mefloquine, halofantrine, and lumefantrine), sesquiterpene trioxanes (artemisinin derivatives), and naphthoquinones (atovaquone) [13, 36, 37]. Identification and lead optimization of a new chemical series as potent antimalarial have several financial, social, and scientific hurdles. These challenges include toxicity, cost, and ethical issues [38–40]. On the other hand, the development of analogues of existing antimalarial drugs is an important and easy way to design new chemotherapeutic interventions, which can tackle the above problems to a greater extent [10, 41]. The development of trioxanes and analogues of 4-aminoquinolines are the best examples of this category [42]. Chloroquine (CQ) analogues synthesized by linking 4,7-dichloroquinoline with monoalkynes, ferroquine, a methalocenic CQ analogue, and 1,2,4-trioxane, which has a similar nucleus to artemisinin, are in clinical trials for the treatment of uncomplicated malaria [43–45].

### 4.2. Analysis of Compounds from Natural Products

Natural products have been used as traditional medicine for thousands of years and have contributed to the arsenal of modern medicine [46–52]. Most of the important and efficacious antimalarial drugs like atovaquone, artemisinin (and its semisynthetic derivatives), clindamycin (a derivative of the natural product lincomycin), erythromycin, and tetracycline have been identified from natural resources only [53–55].

There are several hurdles to overcome to develop antimalarial drugs from natural products, including moderate activity, toxicity, and characterization of physicochemical and biological properties, and most of the time, the isolation of a single drug candidate from the crude will lose the antimalarial potential. However, continuous research is going on worldwide for antimalarial drug development from natural products, and some of them are in clinical trials as well. The development of *Argemone mexicana* as a potent antimalarial is the best recent example of this [56, 57].

### 4.3. Drug Repurposing

Drug repurposing is an emerging trend to develop new chemotherapeutic interventions for many life-threatening diseases like malaria, cancer, tuberculosis, and diabetes [58–65]. It is safe, economic, and less time-consuming to reach the clinical stage as they are already approved for human use, and most of the clinical, pharmacological, pharmacokinetics, and pharmacodynamics information is available.

Several successful efforts have been done in this field, and various molecules have been identified with this approach, particularly for malaria treatment. Itraconazole (an antifungal agent) [66], atorvastatin (widely used to reduce cholesterol levels) [67], lopinavir and tipranavir (HIV protease inhibitors) [68], and the antifungal and antihelminthic compound imidazolopiperazine [68] are the best examples in this context. These molecules have been found to have potent antimalarial activity at different stages and against specific targets of the malaria parasite.

Moreover, several antibiotics like doxycycline, azithromycin, clindamycin, tetracycline, and fosmidomycin are in clinical use as combination partners for malaria treatment. One of the most important antimicrobial agents, sulfadoxine, has the most synergistic antimalarial effects when combined with pyrimethamine. Several countries have adopted this combination in their antimalarial policies [69–71].

### 4.4. Identification of New Drug Targets and Synthesis of Their Selective Inhibitors

Identification of new drug targets for antimalarial drug discovery is a major challenge in the context of multidrug-resistant malaria. Development of drug resistance against one particular antimalarial also reduces the efficacy of closely related drugs called “cross resistance,” which reduces the options for case management to a greater extent [8, 35, 72, 73]. It is therefore necessary to identify some new vital targets and develop new pharmacophores against these targets [74–76].

Whole-genome sequencing of the malaria parasite opened new avenues for the identification of a new drug target [77]. Several key pathways and specific targets like type II fatty acid synthesis (FASII) [78], apicoplast and its various pathways [79–81], shikimate pathway [82], enzymes of the folate pathway [83], and many more from different vital pathways of the malaria parasite are being explored to identify a safe, vital target and its validation, as well as to develop some new and safe therapies against these identified targets [84, 85].

High-throughput in vitro or virtual screening of chemical libraries from different resources is an emerging tool for identification and lead optimization [86]. This approach is not only valuable to develop a new antimalarial drug or combination but also its unique action mechanism will be beneficial to reduce/delay the chances of drug resistance either with existing antimalarial or with newly developed other candidates [87].

### 4.5. Identification of Resistance-Reversal Agents

Antimalarial drug resistance is emerging at a faster rate due to several reasons, like drug abuse, mutations in transporter genes, and mutations in identified target genes. Drug discovery is a time-consuming process that is primarily limited by the duration of lead optimization and clinical trials, their high cost, and strict regulatory rules. Therefore, it is important to optimize the existing antimalarials with proper strategies to reintroduce them into the mainstream.

However, the identification of resistance reversal agents is also a challenging task limited by a lack of action mechanisms and toxicity. Several resistance reversal agents have been identified for most of the traditional drugs like CQ (verapamil, chlorpromazine, promethazine, chlorpheniramine, and citalopram) [88–90], mefloquine, quinine, and quinidine (NP30) [91, 92].

Drug metabolizing enzymes also play an important role in declining efficacy and drug resistance. The excessive metabolism of traditional drugs like mefloquine, quinine, and quinidine may lead to reduced bioavailability, thereby declining the efficacy and inducing the chances of drug resistance. Inhibition of these drug metabolizing enzymes by specific inhibitors can reverse the situation, and the drug might remain effective. However, toxicity might be a major concern for these combinations and should be taken into consideration [93, 94]. Successful research has been done employing this resistance reversal approach. With the resistance reversal action of ketoconazole for mefloquine [95], the combination of clarithromycin with mefloquine/quinine/quinidine against multidrug-resistant malaria [93, 96] has been proved to be beneficial against MDR malaria.

### 4.6. Combination Therapy

Due to the emergence of drug resistance towards most of the antimalarial drugs, WHO banned monotherapy for malaria to reduce the chances of drug resistance. The rationale behind the combination is that if two drugs are used with different modes of action and therefore different resistance mechanisms, then the probability of developing resistance to both drugs at the same cell division is the product of their individual probabilities [97, 98].

Combination therapy with antimalarial drugs is the simultaneous or combined use of two or more blood schizontocidal drugs with independent modes of action. The different biochemical targets enable the combinations to improve therapeutic efficacy as well as delay the development of resistance to the individual components [33, 99–101].

Artemisinin-based combinations (ACTs) are recommended as first-line treatments for uncomplicated falciparum malaria worldwide. These derivatives are fast-acting and active against different stages of the malaria parasite. It is recommended that the other ACT partner drug have a longer half-life due to the shorter half-life of the artemisinin derivatives. The artemisinin derivatives are eliminated rapidly, and the partner drugs are eliminated slowly; therefore, there is complete protection for the artemisinin derivatives [99, 101].

Moreover, resistance/delayed parasite clearance against widely used antimalarial combinations, including ACTs, deteriorates the problem and poses a major threat to the development of new therapies [102–105].

### 4.7. Omics-Based Strategy

Multiomics technologies such as genomics, proteomics, and metabolomics have been widely used in recent years to provide a more holistic perspective of biological mechanisms, functional principles, and dynamics [106]. Omics approaches incorporating high-throughput technology, automation, and data mining have aided in a faster, more reliable, and economical understanding of the molecular pathways and critical proteins required in the parasite's life cycle, and hence the pathogenesis of this disease [107]. The transition to cell-based phenotypic screening has been a significant advancement in antimalarial drug discovery, with noteworthy improvements in the screening of compounds against the asexual blood stage, liver stage, and gametocytes. In vitro development of compound-resistant parasites followed by whole-genome scanning is a common strategy for therapeutic target deconvolution in *Plasmodium falciparum* [107]. This approach has been used to identify or confirm several of the most promising antimalarial drug targets, including translation elongation factor 2 (eEF2) and phenylalanine tRNA synthetase (PheRS). One disadvantage of this strategy is that if a mutant gene is uncharacterized, it may take a lot of time and effort to figure out whether it is a drug target, a drug resistance gene, or just a background mutation. As a result, having high-throughput, functional genomic datasets available can considerably aid target deconvolution. *P. falciparum* genome-wide essentiality mapping and transcriptional analysis of the host and parasite during *P. berghei* liver-stage infection have discovered potentially druggable pathways. The discovery of essential pathways involved in parasite development has been aided by advances in deciphering the epigenomic regulation of the malaria parasite genome. Furthermore, studying the host genome during infection has led to the discovery of new gene candidates linked to severe malaria susceptibility. One of the most successful omics-based approaches for discovering or rediscovering numerous specific novel targets of promising small compounds has been the forward genetics IVIEWGA (in vitro evolution and whole-genome analysis) method [108, 109].

### 4.8. Immunotherapy

Immunotherapy is an emerging and successful strategy to fight against several diseases, including malaria and cancer [110]. Checkpoint blockade immunotherapy is the most notable immunotherapy of recent times, in which monoclonal antibodies (mAbs) are used to break the interaction between immune inhibitory receptor-ligand pairs [110]. This blocking leads to the facilitation of normal immune system function, thereby permitting enhanced immune responses against upregulated ligands. Cytotoxic T-lymphocyte-associated protein 4 (CTLA4), death 1 ligand 1 (PD-L1), T-cell immunoglobulin, mucin domain-containing protein 3 (TIM3), OX40, GITR, and CD69 are the main targets for these mAbs [110].

Recently, one independent preclinical study showed that a multimeric form of PD-L2 fused with the Fc part of immunoglobulin (PD-L2-Fc) was sufficient to reduce the lethal malaria infection and mediate survival following reinfections after several months without additional PD-L2-Fc [111]. Combined inhibition of PD-L1 and LAG3 by mAbs increases the clearance of malaria parasites, facilitates CD4+ T cell function, and increases antibody titres [112]. It has also been shown that inhibition of OX40 signaling also increases helper CD4+ T cells and humoral immunity, thereby increasing parasite clearance during nonlethal malarial infections [113]. Preclinical studies have also shown that inhibition of CTLA4 or PD-L1 increases T cell activation and increases the incidence of cerebral malaria [114].

## 5. Conclusions and Future Directions

Although a marked reduction in malaria incidence and mortality has been reported in recent years due to the introduction of ACTs in antimalarial policy, the development of drug resistance against most of the antimalarials, especially artemisinin derivatives, is an alarming situation. The introduction of new drugs/combinations in the antimalarial arsenal is an immediate need. Based on resources, one or more of the strategies discussed above should be explored for the development of new chemotherapeutic interventions for malaria. Importantly, the use of genomics and omics-based methodologies has resulted in significant breakthroughs in the identification of novel targets in protozoan diseases.

## Figures and Tables

**Table 1:** Important antimalarial drugs and their developmental stages.

Antimalarial	Year of development	Developmental phase
Quinine	1820	In clinical use
Chloroquine	1934	In clinical use
Proguanil	1945	In clinical use
Pyrimethamine	1952	In clinical use
Piperaquine	1966	In clinical use
Lumefantrine	1967	In clinical use
Pyronaridine	1967	In clinical use
Mefloquine	1974	In clinical use
Sulphadoxine	1981	In clinical use
Artemisinin	1972	In clinical use
Atovaquone	1991	In clinical use
Artefenomel	Under development	Phase 1
DSM265	Under development	Phase 1
M5717	Under development	Phase 1
Meplazumab	Under development	Phase 1
MMV688533	Under development	Phase 1
SAR441121	Under development	Phase 1
ZY-19489	Under development	Phase 1
Cipargamin	Under development	Phase 2
DM1157	Under development	Phase 2
MMV390048	Under development	Phase 2
SJ733	Under development	Phase 2
Tafenoquine	Under development	Phase 2
5-ALA HCl with SFC	Under development	Phase 2
Artefenomel-ferroquine	Under development	Phase 2
Imatinib-DHA-piperaquine	Under development	Phase 2
Methylene blue with artemether and lumefantrine	Under development	Phase 2
Ganaplacide with LUM-SDF	Under development	Phase 3
Artemether-lumefantrine	Under development	Phase 4

## Data Availability

Data sharing is not applicable to this article as no new data were created or analyzed in this study.

## References

[1] Nosten F., Richard-Lenoble D., Danis M. (2022). A brief history of malaria. *La Presse Médicale*.

[2] Kelsey A., Stillinger D., Pham T. B., Murphy J., Firth S., Carballar-Lejarazu R. (2020). Global governing bodies: a pathway for gene drive governance for vector mosquito control. *The American Journal of Tropical Medicine and Hygiene*.

[3] Nasir S. M. I., Amarasekara S., Wickremasinghe R., Fernando D., Udagama P. (2020). Prevention of re-establishment of malaria: historical perspective and future prospects. *Malaria Journal*.

[4] Wilson A. L., Courtenay O., Kelly-Hope L. A. (2020). The importance of vector control for the control and elimination of vector-borne diseases. *PLoS Neglected Tropical Diseases*.

[5] Tizifa T. A., Kabaghe A. N., McCann R. S., van den Berg H., Van Vugt M., Phiri K. S. (2018). Prevention efforts for malaria. *Curr Trop Med Rep*.

[6] Zavala F. (2022). RTS,S: the first malaria vaccine. *The Journal of Clinical Investigation*.

[7] Nsanzabana C. (2019). Resistance to artemisinin combination therapies (ACTs): do not forget the partner drug. *Tropical Medicine and Infectious Disease*.

[8] Capela R., Moreira R., Lopes F. (2019). An overview of drug resistance in Protozoal diseases. *International Journal of Molecular Sciences*.

[9] Achan J., Talisuna A. O., Erhart A. (2011). Quinine, an old anti-malarial drug in a modern world: role in the treatment of malaria. *Malaria Journal*.

[10] Belete T. M. (2020). Recent progress in the development of new antimalarial drugs with novel targets. *Drug Design, Development and Therapy*.

[11] Greenwood D. (1995). Conflicts of interest: the genesis of synthetic antimalarial agents in peace and war. *Journal of Antimicrobial Chemotherapy*.

[12] Baird J. K. (2004). Chloroquine resistance in Plasmodium vivax. *Antimicrobial Agents and Chemotherapy*.

[13] Tse E. G., Korsik M., Todd M. H. (2019). The past, present and future of anti-malarial medicines. *Malaria Journal*.

[14] Nixon G. L., Moss D. M., Shone A. E. (2013). Antimalarial pharmacology and therapeutics of atovaquone. *Journal of Antimicrobial Chemotherapy*.

[15] Blanshard A., Hine P. (2021). Atovaquone-proguanil for treating uncomplicated Plasmodium falciparum malaria. *Cochrane Database of Systematic Reviews*.

[16] Roux A. T., Maharaj L., Oyegoke O. (2021). Chloroquine and sulfadoxine-pyrimethamine resistance in sub-saharan africa-A review. *Frontiers in Genetics*.

[17] Milhous W. K. (2001). Development of new drugs for chemoprophylaxis of malaria. *Medecine Tropicale*.

[18] Schlagenhauf P., Adamcova M., Regep L., Schaerer M. T., Rhein H. G. (2010). The position of mefloquine as a 21st century malaria chemoprophylaxis. *Malaria Journal*.

[19] Basco L. K., Ringwald P. (2003). In vitro activities of piperaquine and other 4-aminoquinolines against clinical isolates of Plasmodium falciparum in Cameroon. *Antimicrobial Agents and Chemotherapy*.

[20] Cisse B., Cairns M., Faye E. (2009). Randomized trial of piperaquine with sulfadoxine-pyrimethamine or dihydroartemisinin for malaria intermittent preventive treatment in children. *PLoS One*.

[21] Boonyalai N., Vesely B. A., Thamnurak C. (2020). Piperaquine resistant Cambodian Plasmodium falciparum clinical isolates: in vitro genotypic and phenotypic characterization. *Malaria Journal*.

[22] Ezzet F., van Vugt M., Nosten F., Looareesuwan S., White N. J. (2000). Pharmacokinetics and pharmacodynamics of lumefantrine (benflumetol) in acute falciparum malaria. *Antimicrobial Agents and Chemotherapy*.

[23] Chang C., Lin-Hua T., Jantanavivat C. (1992). Studies on a new antimalarial compound: pyronaridine. *Transactions of the Royal Society of Tropical Medicine and Hygiene*.

[24] Croft S. L., Duparc S., Arbe-Barnes S. J. (2012). Review of pyronaridine anti-malarial properties and product characteristics. *Malaria Journal*.

[25] Antoine T., Fisher N., Amewu R., O’Neill P. M., Ward S. A., Biagini G. A. (2014). Rapid kill of malaria parasites by artemisinin and semi-synthetic endoperoxides involves ROS-dependent depolarization of the membrane potential. *Journal of Antimicrobial Chemotherapy*.

[26] Meshnick S. R., Yang Y. Z., Lima V., Kuypers F., Kamchonwongpaisan S., Yuthavong Y. (1993). Iron-dependent free radical generation from the antimalarial agent artemisinin (qinghaosu). *Antimicrobial Agents and Chemotherapy*.

[27] Lin A. J., Klayman D. L., Ager A. L. (1994). Antimalarial activity of dihydroartemisinin derivatives by transdermal application. *The American Journal of Tropical Medicine and Hygiene*.

[28] Mohanty S., Mishra S. K., Satpathy S. K., Dash S., Patnaik J. (1997). *α*, *β*-Arteether for the treatment of complicated falciparum malaria. *Transactions of the Royal Society of Tropical Medicine and Hygiene*.

[29] Karunajeewa H. A., Manning L., Mueller I., Ilett K. F., Davis T. M. E. (2007). Rectal administration of artemisinin derivatives for the treatment of malaria. *JAMA*.

[30] Myint H. Y., Ashley E. A., Day N. P., Nosten F., White N. J. (2007). Efficacy and safety of dihydroartemisinin-piperaquine. *Transactions of the Royal Society of Tropical Medicine and Hygiene*.

[31] Morris C. A., Duparc S., Borghini-Fuhrer I., Jung D., Shin C. S., Fleckenstein L. (2011). Review of the clinical pharmacokinetics of artesunate and its active metabolite dihydroartemisinin following intravenous, intramuscular, oral or rectal administration. *Malaria Journal*.

[32] WHO (2022). *World Malaria Report*.

[33] White N. J. (2004). Antimalarial drug resistance. *Journal of Clinical Investigation*.

[34] Buyon L. E., Elsworth B., Duraisingh M. T. (2021). The molecular basis of antimalarial drug resistance in Plasmodium vivax. *International Journal for Parasitology: Drugs and Drug Resistance*.

[35] Wicht K. J., Mok S., Fidock D. A. (2020). Molecular mechanisms of drug resistance in plasmodium falciparum malaria. *Annual Review of Microbiology*.

[36] Ashley E. A., Phyo A. P. (2018). Drugs in development for malaria. *Drugs*.

[37] Tibon N. S., Ng C. H., Cheong S. L. (2020). Current progress in antimalarial pharmacotherapy and multi-target drug discovery. *European Journal of Medicinal Chemistry*.

[38] De Rycker M., Baragana B., Duce S. L., Gilbert I. H. (2018). Challenges and recent progress in drug discovery for tropical diseases. *Nature*.

[39] Jamrozik E., de la Fuente-Nunez V., Reis A., Ringwald P., Selgelid M. J. (2015). Ethical aspects of malaria control and research. *Malaria Journal*.

[40] Cheah P. Y., White N. J. (2016). Antimalarial mass drug administration: ethical considerations. *International Health*.

[41] Okombo J., Chibale K. (2018). Recent updates in the discovery and development of novel antimalarial drug candidates. *Medchemcomm*.

[42] Narula A. K., Azad C. S., Nainwal L. M. (2019). New dimensions in the field of antimalarial research against malaria resurgence. *European Journal of Medicinal Chemistry*.

[43] Adoke Y., Zoleko-Manego R., Ouoba S. (2021). A randomized, double-blind, phase 2b study to investigate the efficacy, safety, tolerability and pharmacokinetics of a single-dose regimen of ferroquine with artefenomel in adults and children with uncomplicated Plasmodium falciparum malaria. *Malaria Journal*.

[44] McCarthy J. S., Ruckle T., Djeriou E. (2016). A Phase II pilot trial to evaluate safety and efficacy of ferroquine against early Plasmodium falciparum in an induced blood-stage malaria infection study. *Malaria Journal*.

[45] Wani W. A., Jameel E., Baig U., Mumtazuddin S., Hun L. T. (2015). Ferroquine and its derivatives: new generation of antimalarial agents. *European Journal of Medicinal Chemistry*.

[46] Anand U., Jacobo-Herrera N., Altemimi A., Lakhssassi N. (2019). A comprehensive review on medicinal plants as antimicrobial therapeutics: potential avenues of biocompatible drug discovery. *Metabolites*.

[47] Dziubko N. I., Tolstopiatov B. A., Konovalenko V. F., Chernenko O. D., Redkousova S. F. (1987). [Effect of splenin and zymosan on the immune system of patients with a soft tissue sarcoma]. *Vrachebnoe Delo*.

[48] Das T., Anand U., Pandey S. K. (2021). Therapeutic strategies to overcome taxane resistance in cancer. *Drug Resistance Updates*.

[49] Mohammed M. J., Anand U., Altemimi A. B., Tripathi V., Guo Y., Pratap-Singh A. (2021). Phenolic composition, antioxidant capacity and antibacterial activity of white wormwood (Artemisia herba-alba). *Plants*.

[50] Anand U., Tudu C. K., Nandy S. (2022). Ethnodermatological use of medicinal plants in India: from ayurvedic formulations to clinical perspectives - a review. *Journal of Ethnopharmacology*.

[51] Datta S., Ramamurthy P. C., Anand U. (2021). Wonder or evil?: multifaceted health hazards and health benefits of Cannabis sativa and its phytochemicals. *Saudi Journal of Biological Sciences*.

[52] Halder S., Anand U., Nandy S. (2021). Herbal drugs and natural bioactive products as potential therapeutics: a review on pro-cognitives and brain boosters perspectives. *Saudi Pharmaceutical Journal*.

[53] Weber J. M., Wierman C. K., Hutchinson C. R. (1985). Genetic analysis of erythromycin production in Streptomyces erythreus. *Journal of Bacteriology*.

[54] Nelson M. L., Levy S. B. (2011). The history of the tetracyclines. *Annals of the New York Academy of Sciences*.

[55] Argoudelis A. D., Eble T. E., Fox J. A., Mason D. J. (1969). Biosynthesis of lincomycin. IV. Origin of methyl groups. *Biochemistry*.

[56] Rubio-Pina J., Vazquez-Flota F. (2013). Pharmaceutical applications of the benzylisoquinoline alkaloids from Argemone mexicana L. *Current Topics in Medicinal Chemistry*.

[57] Simoes-Pires C., Hostettmann K., Haouala A. (2014). Reverse pharmacology for developing an anti-malarial phytomedicine. The example of Argemone mexicana. *International Journal for Parasitology: Drugs and Drug Resistance*.

[58] Pushpakom S., Iorio F., Eyers P. A. (2019). Drug repurposing: progress, challenges and recommendations. *Nature Reviews Drug Discovery*.

[59] Hua Y., Dai X., Xu Y. (2022). Drug repositioning: progress and challenges in drug discovery for various diseases. *European Journal of Medicinal Chemistry*.

[60] Begley C. G., Ashton M., Baell J. (2021). Drug repurposing: misconceptions, challenges, and opportunities for academic researchers. *Science Translational Medicine*.

[61] Lundstrom-Stadelmann B., Rufener R., Hemphill A. (2020). Drug repurposing applied: activity of the anti-malarial mefloquine against Echinococcus multilocularis. *International Journal for Parasitology: Drugs and Drug Resistance*.

[62] Nabirotchkin S., Peluffo A. E., Rinaudo P., Yu J., Hajj R., Cohen D. (2020). Next-generation drug repurposing using human genetics and network biology. *Current Opinion in Pharmacology*.

[63] Vijayan K., Wei L., Glennon E. K. K. (2021). Host-targeted interventions as an exciting opportunity to combat malaria. *Chemical Reviews*.

[64] Mullins J. G. L. (2022). Drug repurposing in silico screening platforms. *Biochemical Society Transactions*.

[65] Fontinha D., Moules I., Prudencio M. (2020). Repurposing drugs to fight hepatic malaria parasites. *Molecules*.

[66] Pongratz P., Kurth F., Ngoma G. M., Basra A., Ramharter M. (2011). In vitro activity of antifungal drugs against Plasmodium falciparum field isolates. *Wiener Klinische Wochenschrift*.

[67] Parquet V., Henry M., Wurtz N. (2010). Atorvastatin as a potential anti-malarial drug: in vitro synergy in combinational therapy with quinine against Plasmodium falciparum. *Malaria Journal*.

[68] Nsanzabana C., Rosenthal P. J. (2011). *In vitro* activity of antiretroviral drugs against plasmodium falciparum. *Antimicrobial Agents and Chemotherapy*.

[69] Aguiar A. C., de Sousa L. R. F., Garcia C. R. S., Oliva G., Guido R. V. C. (2019). New molecular targets and strategies for antimalarial discovery. *Current Medicinal Chemistry*.

[70] Aguiar A. C. C., Rocha E. M. D, Souza N. B. D, Franca T. C., Krettli A. U. (2012). New approaches in antimalarial drug discovery and development: a review. *Memorias do Instituto Oswaldo Cruz*.

[71] Kumar A., Chery L., Biswas C. (2012). Malaria in south Asia: prevalence and control. *Acta Tropica*.

[72] Duffey M., Blasco B., Burrows J. N., Wells T. N., Fidock D. A., Leroy D. (2021). Assessing risks of Plasmodium falciparum resistance to select next-generation antimalarials. *Trends in Parasitology*.

[73] Amelo W., Makonnen E. (2021). Efforts made to eliminate drug-resistant malaria and its challenges. *BioMed Research International*.

[74] Bassanini I., Parapini S., Galli C. (2019). Discovery and pharmacophore mapping of a low-nanomolar inhibitor of P. Falciparum growth. *ChemMedChem*.

[75] Saddala M. S., Adi P. J. (2018). Discovery of small molecules through pharmacophore modeling, docking and molecular dynamics simulation against Plasmodium vivax Vivapain-3 (VP-3). *Heliyon*.

[76] Fayyazi N., Esmaeili S., Taheri S. (2020). Pharmacophore modeling, synthesis, scaffold hopping and biological beta- hematin inhibition interaction studies for anti-malaria compounds. *Current Topics in Medicinal Chemistry*.

[77] Akoniyon O. P., Adewumi T. S., Maharaj L. (2022). Whole genome sequencing contributions and challenges in disease reduction focused on malaria. *Biology*.

[78] Vaughan A. M., O’Neill M. T., Tarun A. S. (2009). Type II fatty acid synthesis is essential only for malaria parasite late liver stage development. *Cellular Microbiology*.

[79] Elaagip A., Absalon S., Florentin A. (2022). Apicoplast dynamics during plasmodium cell cycle. *Frontiers in Cellular and Infection Microbiology*.

[80] Low L. M., Stanisic D. I., Good M. F. (2018). Exploiting the apicoplast: apicoplast-targeting drugs and malaria vaccine development. *Microbes and Infection*.

[81] Biddau M., Sheiner L. (2019). Targeting the apicoplast in malaria. *Biochemical Society Transactions*.

[82] Kappes B., Macheroux P., Kappes B. (2013). The shikimate pathway in apicomplexan parasites: implications for drug development. *Frontiers in Bioscience*.

[83] Salcedo-Sora J. E., Ward S. A. (2013). The folate metabolic network of Falciparum malaria. *Molecular and Biochemical Parasitology*.

[84] Qidwai T., Jamal F., Singh S. (2014). Exploring putative molecular mechanisms of human pyruvate kinase enzyme deficiency and its role in resistance against Plasmodium falciparum malaria. *Interdisciplinary Sciences: Computational Life Sciences*.

[85] Batista F. A., Gyau B., Vilacha J. F. (2020). New directions in antimalarial target validation. *Expert Opinion on Drug Discovery*.

[86] Antonova-Koch Y., Meister S., Abraham M. (2018). Open-source discovery of chemical leads for next-generation chemoprotective antimalarials. *Science*.

[87] Guiguemde W. A., Shelat A., Garcia-Bustos J., Diagana T. T., Gamo F. J., Guy R. (2012). Global phenotypic screening for antimalarials. *Chemistry and Biology*.

[88] van Schalkwyk D. A., Egan T. J. (2006). Quinoline-resistance reversing agents for the malaria parasite Plasmodium falciparum. *Drug Resistance Updates*.

[89] Masseno V., Muriithi S., Nzila A. (2009). In vitro chemosensitization of Plasmodium falciparum to antimalarials by verapamil and probenecid. *Antimicrobial Agents and Chemotherapy*.

[90] Evans S. G., Butkow N., Stilwell C., Berk M., Kirchmann N., Havlik I. (1998). Citalopram enhances the activity of chloroquine in resistant plasmodium in vitro and in vivo. *Journal of Pharmacology and Experimental Therapeutics*.

[91] Egan T. J., Kaschula C. H. (2007). Strategies to reverse drug resistance in malaria. *Current Opinion in Infectious Diseases*.

[92] Ciach M., Zong K., Kain K. C., Crandall I. (2003). Reversal of mefloquine and quinine resistance in Plasmodium falciparum with NP30. *Antimicrobial Agents and Chemotherapy*.

[93] Pandey S. K., Dwivedi H., Singh S., Siddiqui W. A., Tripathi R. (2013). Antimalarial interaction of quinine and quinidine with clarithromycin. *Parasitology*.

[94] Gunjan S., Singh S., Chauhan B. (2015). Clarithromycin enhances the antimalarial efficacy of mefloquine via its increased bioavailability and disrupting P. falciparum apicoplast. *Life Sciences*.

[95] Awasthi A., Dutta G. P., Bhakuni V., Tripathi R. (2004). Resistance reversal action of ketoconazole against mefloquine resistance of Plasmodium yoelii nigeriensis. *Experimental Parasitology*.

[96] Tripathi R., Pandey S. K., Rizvi A. (2011). Clarithromycin, a cytochrome P450 inhibitor, can reverse mefloquine resistance in Plasmodium yoelii nigeriensis- infected Swiss mice. *Parasitology*.

[97] Bhagavathula A. S., Elnour A. A., Shehab A. (2016). Alternatives to currently used antimalarial drugs: in search of a magic bullet. *Infect Dis Poverty*.

[98] Rasmussen C., Alonso P., Ringwald P. (2022). Current and emerging strategies to combat antimalarial resistance. *Expert Review of Anti-infective Therapy*.

[99] Nosten F., White N. J. (2007). Artemisinin-based combination treatment of falciparum malaria. *The American Journal of Tropical Medicine and Hygiene*.

[100] White N. J. (2008). Qinghaosu (artemisinin): the price of success. *Science*.

[101] World Health Organization (2010). *Guidelines for the treatment of malaria*.

[102] Lell B., Kremsner P. G., Sturchler D., Lehman L. G., Handschin J., Schmidt-Ott J. R. (1998). Malaria chemotherapy trial at a minimal effective dose of mefloquine/sulfadoxine/pyrimethamine compared with equivalent doses of sulfadoxine/pyrimethamine or mefloquine alone. *The American Journal of Tropical Medicine and Hygiene*.

[103] Faucher J. F., Binder R., Missinou M. (2002). Efficacy of atovaquone/proguanil for malaria prophylaxis in children and its effect on the immunogenicity of live oral typhoid and cholera vaccines. *Clinical Infectious Diseases*.

[104] Davis W. A., Clarke P. M., Siba P. M. (2011). Cost-effectiveness of artemisinin combination therapy for uncomplicated malaria in children: data from Papua New Guinea. *Bulletin of the World Health Organization*.

[105] Phyo A. P., Ashley E. A., Anderson T. J. C. (2016). Declining efficacy of artemisinin combination therapy against P. Falciparum malaria on the Thai-Myanmar border (2003-2013): the role of parasite genetic factors. *Clinical Infectious Diseases*.

[106] Singh R., Singh P. K., Kumar R. (2021). Multi-omics approach in the identification of potential therapeutic biomolecule for COVID-19. *Frontiers in Pharmacology*.

[107] Zhou M., Varol A., Efferth T. (2021). Multi-omics approaches to improve malaria therapy. *Pharmacological Research*.

[108] Cowell A. N., Winzeler E. A. (2019). Advances in omics-based methods to identify novel targets for malaria and other parasitic protozoan infections. *Genome Medicine*.

[109] Luth M. R., Gupta P., Ottilie S., Winzeler E. A. (2018). Using in vitro evolution and whole genome analysis to discover next generation targets for antimalarial drug discovery. *ACS Infectious Diseases*.

[110] Naran K., Nundalall T., Chetty S., Barth S. (2018). Principles of immunotherapy: implications for treatment strategies in cancer and infectious diseases. *Frontiers in Microbiology*.

[111] Crompton P. D., Pierce S. K. (2016). PD-L2 elbows out PD-L1 to rescue T cell immunity to malaria. *Immunity*.

[112] Furtado R., Chorro L., Zimmerman N. (2020). Blockade of LAG-3 in PD-L1-deficient mice enhances clearance of blood stage malaria independent of humoral responses. *Frontiers in Immunology*.

[113] Othman A. S., Franke-Fayard B. M., Imai T. (2018). OX40 stimulation enhances protective immune responses induced after vaccination with attenuated malaria parasites. *Frontiers in Cellular and Infection Microbiology*.

[114] Hafalla J. C. R., Claser C., Couper K. N. (2012). The CTLA-4 and PD-1/PD-L1 inhibitory pathways independently regulate host resistance to Plasmodium-induced acute immune pathology. *PLoS Pathogens*.

